# High Intratumoral *PROS1* Expression Correlates with Improved Survival and Is Associated with Suppressed Oncogenic Signaling in Pancreatic Ductal Adenocarcinoma

**DOI:** 10.3390/ijms27072964

**Published:** 2026-03-25

**Authors:** Teagan Prouse, Rinku Majumder, Samarpan Majumder

**Affiliations:** 1 Department of Interdisciplinary Oncology, Louisiana State University Health Sciences Center, New Orleans, LA 70112, USA; tprous@lsuhsc.edu; 2 Department of Genetics, Louisiana State University Health Sciences Center, New Orleans, LA 70112, USA

**Keywords:** pancreatic ductal adenocarcinoma, venous thromboembolism, *PROS1*, *SNAI2*, *MMP2*, hazard ratio meta-analysis, Spearman correlation coefficient meta-analysis, epithelial-to-mesenchymal transition

## Abstract

Pancreatic ductal adenocarcinoma (PDAC) is a highly aggressive malignancy with a five-year survival rate of approximately 13%. Patients with PDAC also have an elevated incidence of venous thromboembolism (VTE), despite prophylactic anticoagulation. Thus, there is an urgent need for therapeutic strategies that target tumor progression and hypercoagulability. Protein S (PS), a physiological anticoagulant encoded by the *PROS1* gene, has recently been shown to inhibit PDAC growth in preclinical models. To further examine the physiological relevance of intratumoral PS in PDAC, we performed a meta-analysis of four independent PDAC patient cohorts obtained from cBioPortal. Patients were stratified based on low versus high intratumoral *PROS1* expression based on below- and above-average mean expression, overall survival, and gene expression of select pro-growth genes, implementing a fixed-effects model. High intratumoral *PROS1* expression was associated with a 41.8% reduction in the risk of death compared with low *PROS1* expression (pooled hazard ratio = 0.581) within 30 months of diagnosis from survival data in three cohorts. Elevated *PROS1* expression correlated with marked downregulation of key genes implicated in PDAC invasion and metastasis, including *MMP2* and *SNAI2,* in all four cohorts. Collectively, these findings suggest that *PROS1* is a potential prognostic biomarker and molecular regulator in PDAC and thus support further investigation into the dual role of PS in tumor progression.

## 1. Introduction

Pancreatic cancer is a highly aggressive malignancy characterized by rapid progression and a poor prognosis, ranking among the deadliest human cancers [1]. More than 90% of pancreatic cancer cases are classified as pancreatic ductal adenocarcinoma (PDAC) [2]. Pancreatic cancer commonly presents asymptomatically during its early stages, which leads to late-stage diagnosis. This late presentation is a critical factor in its high mortality rate, characterized by a five-year survival rate of approximately 13% [3,4,5]. Pancreatic cancer is currently the third-leading cause of cancer-related death in the United States [6].

Pancreatic ductal adenocarcinoma (PDAC) is also constrained by limited therapeutic options, resulting in a substantial burden of morbidity and mortality [1]. Therapeutic options for PDAC remain limited. Surgical resection is the only potentially curative intervention; however, fewer than 20% of patients are eligible at diagnosis, and recurrence rates remain high even after complete resection [7].

Furthermore, PDAC demonstrates significant metabolic reprogramming, EMT plasticity, and survival signaling, which significantly contribute to the aggressive nature and poor prognosis of the disease [8]. Pancreatic cancer cells reprogram to drive aerobic glycolysis, as well as glutamine uptake for anabolic processes [9,10]. The metabolic plasticity demonstrated by PDAC contributes to drug resistance phenotypes, which have suggested metabolic shifts as potential actionable therapeutic targets [11]. Additionally, the epithelial-to-mesenchymal transition (EMT) is a central driver of PDAC, which enhances therapy resistance and tumor progression and metastasis [12]. Recent studies have shown that EMT promotes therapy resistance to *KRAS*-targeted treatment [13]. EMT regulatory networks through TGF-β, Wnt, and Notch signaling represent a significant therapeutic target [14]. Both metabolic and EMT signaling pathways integrate with survival signaling pathways (MAPK/ERK, PI3K/AKT) to promote PDAC cell survival under therapeutic stress [9]. Thus, crosstalk in the tumor microenvironment significantly impacts PDAC survival, highlighting the translational relevance of targeting and understanding metabolic-EMT-survival signaling axis interactions.

Beyond its aggressiveness, PDAC is associated with a profound hypercoagulable state. Among all solid malignancies, PDAC carries the highest risk of venous thromboembolism (VTE), with reported incidence rates as high as 57% [15,16,17]. Notably, VTE development in PDAC patients is associated with significantly reduced survival [15,18], underscoring the clinical intersection between thrombosis and PDAC progression.

Current anticoagulation guidelines for pancreatic cancer recommend thromboprophylaxis with direct oral anticoagulants (DOAC) or low-molecular-weight heparin (LMWH) for patients not considered at high risk of bleeding and who are receiving outpatient chemotherapy [19,20]. Among DOAC-treated PDAC cohorts, apixaban is the most prescribed agent (88%), followed by rivaroxaban (12%) [16]. Both apixaban and rivaroxaban are direct inhibitors of factor Xa [21]. Even though these medications are standard of care, a study found that rivaroxaban does not affect the growth of human pancreatic tumors in mice [22]. These observations highlight an unmet clinical need for therapeutic strategies that concurrently mitigate thrombotic risk while targeting tumor progression.

Protein S (PS), a vitamin K-dependent plasma glycoprotein, has emerged as a compelling negative regulator of PDAC tumor growth. Canonically, PS is an essential physiological anticoagulant with several physiological functions. In coagulation, PS acts as a cofactor for activated protein C (APC), enhancing the inactivation of coagulation factors Va and VIIIa [23,24,25]. Protein S is also a cofactor of Tissue Factor Pathway Inhibitor (TFPI) and inhibits factor Xa with TFPI by 4 to 10-fold [26,27,28]. Independently, PS directly inhibits factor IXa (FIXa) activity both in the presence and absence of FVIIIa [29,30] and regulates the propagation phase of coagulation, independent of APC [31].

Beyond its anticoagulant role, PS has been implicated in hypoxia-responsive signaling. Stabilization of hypoxia-inducible factor 1 (HIF-1) in the liver—a physiological response to hypoxia—is associated with reduced *PROS1* expression and lower circulating PS levels [32]. Protein S also acts as a ligand for TAM receptors (Tyro3, Axl, and Mer), a family of receptor tyrosine kinases associated with apoptotic clearance, inflammation resolution, and hemostasis regulation [33].

In addition to the role of PS in hypoxia, PS has also been shown to exert direct antitumor effects on three PDAC cell lines (PANC-1, MiaPaCa-2, and BxPC-3). In these cell lines, PS was shown to be a natural promoter of apoptosis, and more aggressive PDAC cell lines exhibited lower endogenous PS expression and secretion compared to less aggressive cell lines. Furthermore, PS overexpression reduces the survival and proliferation of PDAC cells [34].

In vivo studies further support the antitumor potential of PS. In immunocompromised mice bearing PANC-1 xenografts, systemic PS administration resulted in a dose-dependent reduction in tumor volume, with treated tumors appearing smaller and less vascularized than controls. Importantly, no significant differences in tumor weight or evidence of systemic toxicity were observed between treatment groups [35]. Although no published longitudinal study has been objectively designed to assess free or bound Protein S (PS) levels in PDAC patients, Lindahl et al. observed decreasing PS levels with increasing disease severity [36].

To better define the molecular relevance of PS in human pancreatic cancer, this study investigates the clinical and molecular significance of intratumoral *PROS1* expression in human PDAC using meta-analysis of publicly available cohorts on cBioportal. By integrating survival outcomes with gene expression profiling, we aim to elucidate *PROS1*-associated signaling influences tumor aggressiveness and metastatic potential, providing a foundation for future investigations into PS as a dual therapeutic target in PDAC progression and thrombosis.

## 2. Results

### 2.1. High Intratumoral PROS1 Expression Is Associated with Improved Survival in PDAC

To evaluate the clinical relevance of intratumoral *PROS1* in PDAC, we first assessed whether differential expression of *PROS1* impacts patient survival. Tumors were stratified into low and high *PROS1* expression groups across three independent patient cohorts, and pooled survival analyses were performed to determine associations between *PROS1* expression and overall outcomes. Only three cohorts were implemented for the survival analyses due to the absence of survival data in the fourth cohort. These analyses provide a foundation for subsequent investigations into the molecular pathways associated with *PROS1* in PDAC.

Results summarized in Table 1 include a combined sample size of 397 patients. Specifically, patients in the high *PROS1* group exhibited a pooled hazard ratio of 0.581, corresponding to an approximately 41.8% reduction in the risk of death relative to those in the low *PROS1* group. This association was both clinically meaningful and statistically significant, with a pooled *p*-value of 0.000135.

Individually, the hazard ratios across the cohorts were consistent: TCGA GDC (HR = 0.620, 95% CI 0.394–0.975), TCGA PanCancer (HR = 0.586, 95% CI 0.377–0.918), and CPTAC Cell 2021 (HR = 0.507, 95% CI 0.277–0.926), with cohort weights of 38%, 40%, and 22%, respectively. These results underscore the robustness of the association between high *PROS1* expression and improved survival across multiple patient datasets, suggesting that *PROS1* may serve as a valuable prognostic marker in PDAC.

When modeled as a continuous variable, *PROS1* expression was not significantly associated with overall survival in any of the three cohorts, with HR per unit increase ~1 for TCGA and GDC, and a modest, non-significant trend in CPTAC Cell 2021 of HR per unit increase ~0.9. This suggests that the dichotomized survival difference may not reflect a linear dose–response relationship on *PROS1* expression (Supplemental Table S1).

### 2.2. Differential Expression of Key PDAC-Associated Genes According to Intratumoral PROS1 Expression

To explore potential molecular mechanisms underlying the survival advantage associated with high *PROS1* expression, we analyzed a select set of genes involved in critical PDAC pathways rather than performing an unbiased genome-wide screen among four cohorts. This targeted approach was designed to identify specific signaling pathways potentially modulated by intratumoral *PROS1* expression and to generate testable hypotheses for subsequent in vitro and in vivo studies. Genes associated with TAM receptor signaling, nuclear proliferation, oncogenic signaling, immunosuppression, hypoxia, metabolic reprogramming, epithelial–mesenchymal transition (EMT), invasion, and stemness were selected for analysis.

Between-cohort heterogeneity was evaluated with Cochran’s Q statistic, Q *p*-value, and the I^2^ metric for each gene. Across 49 genes analyzed, all Q tests were nonsignificant (*p* < 0.05), indicating that no variability exists beyond expected sampling error. Only six genes demonstrated Q values exceeding degrees of freedom (df = 3), and the I^2^ values exceeded 26% for just five genes, consistent with low-to-moderate heterogeneity by conventional thresholds (Supplemental Table S2). As between-cohort variability was minimal and consistent with sampling variation, a fixed-effects model was considered appropriate for pooled analyses.

Among the genes evaluated, four exhibited a lower magnitude change in the Spearman correlation coefficient (Δρ) in tumors with high *PROS1* expression compared with low *PROS1* expression: *SLC2A1*, *NOTCH1*, *HK2*, and *TWIST1* (Table 2). *SLC2A1* encodes the glucose transporter GLUT1 [37]; *HK2* encodes hexokinase II, a key glycolytic enzyme [38]; *TWIST1* encodes Twist-related protein 1, a transcription factor implicated in EMT [39]; and *NOTCH1* encodes the Notch1 receptor, a key mediator of developmental and oncogenic signaling pathways that influence proliferation and stemness [40].

Six genes demonstrated an intermediate magnitude Δρ in association with high intratumoral *PROS1* expression: *CDH2*, *TNF*, *TGFB1*, *CA9*, *MMP7*, and *MMP9* (Table 2). These genes encode N-cadherin, tumor necrosis factor-α, transforming growth factor-β1, carbonic anhydrase IX, and matrix metalloproteinases 7 and 9, respectively, and are collectively involved in EMT, immunosuppression, hypoxia adaptation, angiogenesis, and tumor invasion [41,42,43,44,45].

Notably, the highest magnitude Δρ was observed for *MMP2* and *SNAI2*, which encode matrix metalloproteinase 2 and the EMT-associated transcription factor SLUG (Snail family transcriptional repressor 2), respectively (Table 2) [46,47]. These findings suggest that elevated intratumoral *PROS1* expression is inversely correlated with genes associated with molecular pathways involved in metabolic adaptation, EMT, invasion, and tumor–microenvironment interactions in PDAC.

## 3. Discussion

Pancreatic ductal adenocarcinoma is a highly aggressive malignancy, characterized by a poor prognosis [1]. In addition to aggressive tumor biology, PDAC is uniquely associated with a markedly elevated risk of venous thromboembolism (VTE), which contributes substantially to patient mortality [48]. Even though DOACs are the standard-of-care prescribed medication to treat hypercoagulability risk in PDAC patients [19,20], these agents do not address tumor progression. Therapeutic strategies capable of simultaneously modulating coagulation and tumor biology would therefore represent a meaningful advance in PDAC care.

Recent studies conducted by Pilli et al. and Prouse et al. show that PS inhibits PDAC cell growth in vitro and reduces tumor burden in vivo [34,35]. Thus, further investigation was taken to determine if intratumoral PS expression impacts survival in PDAC patients, given its inhibitory effect in vitro and in vivo. Analysis of RNA-sequencing data from human studies paired with evaluation of survival data showed that patients with high *PROS1*-expressing tumors experience a 41.8% lower hazard rate of death up to 30 months after pancreatic cancer diagnosis compared to patients with low *PROS1*-expressing tumors.

These findings support a protective association between intratumoral *PROS1* expression and early survival in PDAC, as assessed by a dichotomized survival model. However, this was not observed when *PROS1* was modeled as a continuous variable, as discussed earlier, suggesting that the observed prognostic signal may depend on expression stratification rather than a consistent linear effect. Accordingly, the prognostic relevance of *PROS1* should be interpreted in the context of model specification.

This discordance does not necessarily invalidate the biological relevance of *PROS1* in cancer prognosis; rather, it underscores the inherent complexity of translating molecular signals into linear survival models. Cancer is a complex, dynamic, highly mutable disease shaped by a constantly evolving tumor microenvironment, clonal heterogeneity, and context-dependent gene regulatory networks. In this setting, the relationship between a single gene’s expression and patient outcome is rarely linear or uniform across the full expression spectrum. Instead, survival differences may emerge at the expression threshold, where a critical biological transition occurs rather than scaling proportionally across all expression levels.

To explore potential mechanisms underlying this survival advantage, we examined the expression of select genes essential to PDAC growth, metabolism, invasion, and immune modulation. Notably, none of the analyzed genes were upregulated in association with high *PROS1* expression, whereas multiple genes demonstrated graded magnitude Δρ, suggesting a suppressive role for *PROS1* across several oncogenic pathways.

The *SLC2A1* gene encodes GLUT1, which is part of a major family of transmembrane receptors that mediate the movement of glucose intracellularly. The GLUT-1 transporter is widely distributed across the body and is necessary for low-level basal glucose uptake in all cells [49]. GLUT-1 overexpression has been associated with HIF-1 signaling and loss of function of p53 [50,51]. Hexokinase II similarly promotes the proliferation, migration, and invasion of PDAC cells under hypoxic conditions [38]. Changes in the Δρ magnitude of these metabolic regulators in high *PROS1*-expressing tumors suggest that PS may influence metabolic adaptation within the PDAC tumor microenvironment.

High *PROS1* expression was also associated with reduced expression of *NOTCH1* and *TWIST1.* In pancreatic cancer, Notch has been reported to crosstalk in signaling pathways that promote PDAC growth and development, including TGF-β [52], MEK/ERK [53], Hedgehog [54], and Wnt signaling [55]. Overall, Notch is a critically important signaling molecule that induces pancreatic cancer growth and promotes EMT, although the exact molecular mechanism by which it does is not known [40]. Twist1 is key pro-growth transcription factor that regulates invasion, metastasis, and also promotes PDAC-associated muscle cachexia [39]. Furthermore, reduced expression of the *CDH2* gene, encoding the EMT protein N (neuronal)-cadherin, indicates potential impairment of EMT [56,57]. Decreased expression of *NOTCH1*, *TWIST1*, and *CDH2* suggests that *PROS1* may modulate EMT-related transcriptional signaling.

Furthermore, a lower magnitude Δρ of *TNF* and *TGFB1* suggests modulation of the immunosuppressive tumor microenvironment. TNF-α is derived from tumor-associated macrophages in PDAC and promotes immunosuppression in PDAC [42], while high levels of TGFβ are critical for immune evasion, EMT induction, invasion, and metastasis [43,58,59]. Lower expression of these cytokines in high *PROS1* tumors implies reduced immunosuppressive and pro-invasive signaling.

High *PROS1* expression was also associated with decreased *CA9* expression, a hypoxia-inducible enzyme critical for tumor adaptation to acidosis and extracellular matrix interactions [60,61,62,63]. Given that *CA9* is a well-established HIF-1 target, these findings further link *PROS1* expression to hypoxia-responsive signaling pathways [44,64]. Prouse et al. demonstrated that the tumor-associated carbonic anhydrase 9 (*CA9*) is attenuated by PS supplementation in vivo [35].

The highest magnitude Δρ of expression was observed for *MMP2* and *SNAI2*. Matrix metalloproteinases MMP-2, MMP-7, and MMP-9 facilitate extracellular matrix degradation, angiogenesis, and metastatic dissemination in PDAC [45,46,65,66,67,68,69,70]. SLUG, encoded by *SNAI2*, is a master regulator of EMT, chemoresistance, invasion, and metastatic competence in pancreatic cancer [71,72]. Suppression of these genes in high *PROS1*-expressing tumors highlights a potential role for PS in restraining invasive and metastatic signaling pathways in PDAC. Overall, these changes reflect alterations in co-expression network behavior rather than illustrating direct regulatory effects of *PROS1*. Further in vivo and in vitro characterization of *PROS1* signaling pathways in PDAC is the next step toward understanding the direct inhibitory effects of high and low intratumoral *PROS1* expression on these key regulatory pathways.

Notably, the observed magnitude Δρ of expression of several EMT-, hypoxia-, and invasion-associated genes should be interpreted as associations derived from a curated set of candidate markers rather than evidence of transcriptome-wide pathway suppression. Accordingly, these findings suggest potential relationships between *PROS1* expression and selected biological processes but do not establish coordinated regulation of entire oncogenic pathways.

While our findings position *PROS1* as a potential prognostic biomarker and molecular regulator in PDAC, potentially influencing coagulation–tumor signaling axes, the clinical translation of these transcriptomic insights remains a key priority. Given that *PROS1* encodes a secreted protein with established roles in anticoagulation and emerging implications in tumor-immune dynamics, *PROS1*-associated molecular signatures (e.g., pathway modulation or expression levels) could be monitored non-invasively via liquid biopsy platforms. Recent advances in AI-driven analysis of extracellular vesicles (EVs) using mid-infrared (Fourier-transform infrared, FTIR) spectroscopy offer a promising, label-free approach for detecting tumor-associated molecular alterations in plasma-derived samples [73]. For instance, autoencoder-based modeling of ATR-FTIR spectra has enabled the extraction of biologically relevant latent features from EVs, discriminating against cancer (e.g., hepatocellular carcinoma) from non-malignant controls with performance comparable to established markers [73,74]. Although initially demonstrated in other malignancies, this methodology’s non-invasive nature, rapid turnaround, and potential for serial sampling make it highly suitable for dynamic monitoring of *PROS1*-related pathway activity or broader coagulation–tumor interactions in PDAC patients. Integrating such AI-enhanced spectroscopic profiling with transcriptomic data could validate *PROS1* signatures in larger cohorts, facilitate early detection, treatment response assessment, and complement existing biomarkers (e.g., CA19-9).

### Limitations

First, the analyses conducted are based on retrospective, publicly available patient datasets, which may vary in sample processing, sequencing platforms, and clinical annotation. Furthermore, due to a lack of treatment data and inconsistent and/or absent patient staging data in each cohort, survival analyses could not be adjusted for treatment type, time treatment began, and patient stage. Furthermore, molecular subtype and race data were also limited or incomplete in each of the three cohorts used to conduct survival analyses. While each patient’s age and sex were entered for all three cohorts (Supplemental Table S3), the impact of sex and age on the probability of pancreatic cancer and/or treatment success is multifaceted, and findings vary significantly between studies [75,76,77]. Furthermore, the survival analysis incorporated a 30-month truncation, which may exclude a small number of long-term survivors who represent legitimate right-tail observations in survival distributions. Although the number of long-term survivors was small and unlikely to influence the primary findings, the absence of a formal sensitivity analysis including all survival times represents a limitation and should be considered when interpreting results.

Second, the study is correlative, and although associations between high *PROS1* expression and improved survival, as well as attenuated oncogenic gene expression, were observed, causality cannot be inferred. Third, only a curated panel of PDAC-relevant genes was analyzed, and broader transcriptomic or proteomic effects of *PROS1* remain uncharacterized. Conclusions regarding direct or indirect inhibitory effect of *PROS1* on certain signaling pathways are thus limited due to the absence of protein-level validation, lack of immune infiltration quantification, and no experimental validation of *PROS1* signaling networks. Despite these limitations, the present study establishes *PROS1* as a candidate prognostic biomarker and provides a framework for future translational investigations into its dual role in PDAC progression and hypercoagulability.

## 4. Materials and Methods

### 4.1. cBioPortal Cohorts

All gene expression and survival data for pancreatic ductal adenocarcinoma were obtained from cBioPortal for Cancer Genomics (https://www.cbioportal.org, accessed on 1 December 2025) [78]. A total of four cohorts were implemented: TCGA GDC, CPTAC GDC 2025, TCGA PanCancer Atlas, and CPTAC Cell 2021. Because analyses were restricted to a single cancer type and did not involve integration of external transcriptomic datasets, additional batch correction was not applied. All dichotomized analyses were conducted strictly within-cohort rather than on pooled transcriptomic data across the four cohorts, thereby limiting susceptibility to cross-study technical confounding. Only standardized summary measures were combined across datasets for comparative purposes.

### 4.2. Definition of High and Low Intratumoral PROS1 Expression

For each cohort independently, the cohort-specific average *PROS1* expression is calculated using filtered data, with the top 5% and lower 5% of outliers removed. High *PROS1* expression will be defined as samples with a value ≥ the cohort mean, and low *PROS1* expression will be defined as samples with a value < the cohort mean, as previously discussed. This cut-off is appropriate since the *PROS1* expression data show an approximately normal or near-normal distribution, and this method ensures that the “high” group genuinely represents the above-average values rather than merely the upper half of the ranked data. This allows for biologically interpretable comparisons, which is advantageous when investigating *PROS1* expression in conjunction with the differential expression of select tumor-associated genes, hypoxia markers, or clinical features. Furthermore, absolute expression thresholds are not established for *PROS1*, and several prognostic biomarker studies similarly rely on study-specific distribution-based thresholds to stratify continuous expression values into discrete groups for survival and differential analyses. The mean is an accepted cohort-based approach when data are normally distributed, as in the case of the *PROS1* expression data after outlier removal [79,80].

Furthermore, Cox regression analysis was performed to model *PROS1* as a continuous variable to determine whether the survival association reflects a dose–response relationship. Hazard ratios were 95% confidence intervals were calculated, and statistical significance was defined as a two-sided *p* < 0.05.

### 4.3. Pooling of Hazard Ratios: Survival of Patients with High vs. Low Intratumoral PROS1 Expression

Due to a lack of survival data from the CPTAC GDC 2025 cohort, only the TCGA GDC, TCGA PanCancer, and CPTAC Cell 2021 cohorts were used to determine hazard ratios (HR). Hazard ratios were calculated according to GraphPad Prism v.10 software, and statistically significant (*p* = 0.05) log-rank curves were generated of high and low *PROS1* expression for each cohort individually. Extreme outliers (patients noted as having 0 months or >30 months of overall survival) were removed from each survival dataset. The 30-month truncation was conducted due to a few patients surviving past 30 months, which would have unjustly impacted data skew. Furthermore, PDAC has a mortality rate of nearly 80% within the first year of diagnosis [81]. Another study showed that the median survival time after PDAC diagnosis is about 4 months, with only 19% of patients surviving past one-year on average [82]. A complete demographics table demonstrating the number of patients, the age range of the patients, and the total number of patients of each sex in each cohort is shown in Supplemental Table S3.

A fixed-effects model is justified for the survival analysis. When the number of studies is small (k ≤ 5), estimation of between-study variance in random-effects models is statistically unstable and may yield imprecise or biased results [83,84], and heterogeneity metrics such as I^2^ are known to perform poorly in small meta-analyses [85]. The Cochrane Handbook cautions that random-effects variance estimates are unreliable when few studies are available [86].

Conceptually, random-effects models assume that each cohort estimates a different underlying true effect [87]. In the present study, all cohorts represent the same disease entity and applied consistent survival modeling, supporting the assumption of a common underlying effect. Given the absence of statistically significant heterogeneity, low I^2^ estimates, and the known instability of between-study variance estimation with only three cohorts, a fixed-effects meta-analytic framework was selected as the most statistically appropriate and methodologically justified approach.

Furthermore, we did not formally test proportional hazards assumptions, as our primary objective was to estimate overall associations between *PROS1* expression and survival rather than to model time-dependent effects. Given the use of single-covariate Cox models and consistent directional findings across cohorts, we considered the Cox model an appropriate summary measure.

From all cohorts, the reported Mantel–Haenszel hazard ratio and corresponding 95% confidence interval were transformed to a natural logarithmic scale. The standard error of the log-transformed standard error (assuming 95% confidence interval) was derived with the formula $SE=\frac{ln(\mathrm{Upper}\;\mathrm{CI})-ln(\mathrm{Lower}\;\mathrm{CI})}{2\times 1.96}$.

Using an inverse-variance weighted fixed-effects model, the pooled effect estimates were calculated on the log-HR scale. The pooled log-HR was computed by weight and exponentiated to obtain the pooled HR, as done with the 95% CI of the pooled HR. The two-tailed *p*-values of each HR were pooled via Stouffer’s Z-method, a weighted method of pooling. All analyses were performed in Microsoft Excel and GraphPad Prism v.10.

### 4.4. Individual Cohort Level Spearman Correlation Analysis

The nonparametric Spearman rank correlation coefficient was calculated to assess associations between gene expression levels at high and low *PROS1* expression levels of all four cohorts. For each cohort independently, the cohort-specific average *PROS1* expression is calculated using filtered data, with the top 5% of outliers removed. High *PROS1* expression is defined as samples with a value ≥ the cohort mean, and low *PROS1* expression is defined as samples with a value < the cohort mean, as previously discussed.

Note also that the correlation coefficient is interpreted differently in biology. In Table 3, the correlation coefficient range between 0.3 and 0.5 is most appropriate for the correlations discussed below, since *PROS1* expression levels and various markers and variables only explain a modest portion of variation, rather than the majority [88,89].

### 4.5. Pooling of Correlation Coefficients, Confidence Interval, and p-Values

Spearman correlation coefficients (ρ) from all four individual cohorts were transformed to Fisher’s z’ values using the formula $z^{\prime }=\frac{1}{2}ln\left(\frac{1+r}{1-r}\right)$ to stabilize variance, and z’ values were pooled across four independent cohorts with inverse-variance weighting, where weights were defined as sample size (*n*) − 3. The pooled Fisher’s z’ value was converted to a Spearman correlation coefficient with the inverse transformation $r=\frac{e^{2z^{\prime }}-1}{e^{2z^{\prime }}+1}$. Correlation coefficients of ±1 were excluded due to undefined variance under the Fisher transformation.

The standard error of the pooled Fisher’s z’ values was calculated as $\sqrt{1/\sum (n_{i}-3)}$, assuming a 95% confidence interval. Confidence intervals were not pooled directly, and a single interval was calculated from the pooled standard error.

Furthermore, the two-tailed *p*-values from the Spearman correlation coefficients across independent cohorts were converted to Z-scores and combined using Stouffer’s Z-method, assuming weight as n−3 to preserve the effect direction and precision. Combined Z-scores were then converted into two-tailed *p*-values.

Weighting by (n−3) was applied to the effect size and *p*-value pooling procedures to ensure that larger, more precise cohorts contributed proportionally greater to pooled estimates. A heterogeneity assessment was performed using Cochran’s Q and I^2^ metric for each gene. All analyses were performed in Microsoft Excel and GraphPad Prism v.10.

### 4.6. Classification of Direction of Change

Direction of change was defined with conservative effect-size thresholds based on the difference between high and low *PROS1* expression states for each gene $\left(∆\rho =\rho_{high}-\rho_{low}\right)$. This approach is derived from McKenzie et al., where differential correlation is a direct comparison of correlation coefficients between two distinct conditions. Computation of the change between Spearman ρ values, and thus testing ρ, is analogous to the empirical computation of ρ values in permutation tests in significantly larger data sets [90]. Thus, a formalized framework of evaluation allows appropriate differentiation of key gene expression between tumors in a low v. high *PROS1* state.

Furthermore, consideration was given to the correlation coefficient and appropriate interpretation in terms of the relationship. Given that correlation coefficients < 0.3 are considered to have no or a weak relationship, gene correlations beneath that threshold under high or low *PROS1* conditions were considered to have independent expression [88,89]. Thus, only correlation coefficients that were ≥0.3 in magnitude in either low and/or high *PROS1* expression conditions can be interpreted appropriately with ∆ρ criteria. Thus, conservative associations were classified as follows in Table 4.

## 5. Conclusions

Collectively, these findings demonstrate *PROS1* as a potential regulator of PDAC biology, linking high intratumoral expression to improved survival and correlating *PROS1* expression to attenuation of metabolic, hypoxic, immunosuppressive, and EMT-associated pathways. By integrating bioinformatics analyses with mechanistic insights from existing literature, this study highlights candidate signaling pathways through which PS may influence tumor growth, invasion, and the hypercoagulable tumor microenvironment. These results provide a framework for future experimental studies to define the molecular mechanisms of PS and explore its potential as a dual-function therapeutic target in PDAC.

## Figures and Tables

**Table 1:** Summary of pooled hazard ratio data for overall survival in PDAC patients stratified by intratumoral *PROS1* expression.

Study	Sample Size	HR (Mantel–Haenszel)	95% CI (Lower–Upper)	Weight
TCGA GDC	143	0.620	0.394–0.975	38%
TCGA PanCancer	142	0.586	0.377–0.918	40%
CPTAC Cell 2021	112	0.507	0.277–0.926	22%
Pooled	397	0.581	0.438–0.724	100%

**Table 2:** Summary of correlational meta-analysis. The Spearman ρ-value at high and low *PROS1* expression states, the Δρ from high to low *PROS1* expression state, the direction of change, the pooled *p*-value for low and high *PROS1* expression states, the significance, and the biological interpretation of the data.

Gene	ρ (Low *PROS1*)	ρ (High *PROS1*)	Δρ (High–Low)	Direction of Change	Pooled *p*-Value (Low/High)	Significance	Biological Interpretation
***CASP9***	0.230	0.408	0.178	Negligible	3.75 × 10^−4^/5.96 × 10^−7^	Yes	Independent correlation
***AKT1***	0.266	0.357	0.090	Negligible	2.09 × 10^−5^/3.63 × 10^−7^	Yes	Independent correlation
***CD8A***	0.348	0.407	0.059	Negligible	4.15 × 10^−9^/3.54 × 10^−7^	Yes	Independent correlation
***CDH1***	0.290	0.333	0.043	Negligible	1.30 × 10^−4^/3.83 × 10^−6^	Yes	Independent correlation
***MAPK1***	0.346	0.384	0.038	Negligible	2.93 × 10^−7^/3.10 × 10^−8^	Yes	Independent correlation
***HIF3A***	0.230	0.268	0.037	Negligible	3.19 × 10^−4^/6.87 × 10^−5^	Yes	Independent correlation
***IL1B***	0.276	0.288	0.012	Negligible	8.04 × 10^−6^/9.64 × 10^−5^	Yes	Independent correlation
***HIF1A***	0.256	0.261	0.005	Negligible	1.51 × 10^−5^/2.28 × 10^−4^	Yes	Independent correlation
***PFKFB4***	−0.296	−0.295	0.001	Negligible	5.66 × 10^−6^/4.42 × 10^−5^	Yes	Independent correlation
***CASP3***	0.323	0.323	0.000	Negligible	6.12 × 10^−7^/9.13 × 10^−6^	Yes	Independent correlation
***E2F1***	−0.359	−0.370	−0.011	Negligible	7.95 × 10^−8^/1.66 × 10^−7^	Yes	Independent correlation
***PIK3CA***	0.388	0.377	−0.011	Negligible	2.33 × 10^−9^/1.31 × 10^−7^	Yes	Independent correlation
***PTK2***	0.314	0.300	−0.014	Negligible	1.13 × 10^−5^/1.12 × 10^−5^	Yes	Independent correlation
***NRAS***	0.304	0.283	−0.021	Negligible	2.64 × 10^−6^/3.41 × 10^−5^	Yes	Independent correlation
***SNAI1***	0.306	0.271	−0.034	Negligible	4.33 × 10^−7^/3.15 × 10^−4^	Yes	Independent correlation
***HRAS***	−0.273	−0.319	−0.046	Negligible	5.54 × 10^−5^/6.95 × 10^−6^	Yes	Independent correlation
***MERTK***	0.377	0.323	−0.054	Negligible	3.45 × 10^−10^/6.76 × 10^−6^	Yes	Independent correlation
***TYRO3***	0.346	0.283	−0.064	Negligible	4.06 × 10^−7^/6.25 × 10^−5^	Yes	Independent correlation
***PDK1***	0.338	0.265	−0.073	Negligible	3.98 × 10^−6^/2.54 × 10^−5^	Yes	Independent correlation
***STAT3***	0.343	0.264	−0.079	Negligible	1.83 × 10^−7^/1.64 × 10^−4^	Yes	Independent correlation
***PFKFB3***	0.363	0.282	−0.081	Negligible	7.89 × 10^−8^/1.00 × 10^−4^	Yes	Independent correlation
***CD4***	0.429	0.346	−0.083	Negligible	2.56 × 10^−10^/2.41 × 10^−6^	Yes	Independent correlation
***EPAS1***	0.379	0.296	−0.084	Negligible	3.17 × 10^−8^/1.97 × 10^−5^	Yes	Independent correlation
***PECAM1***	0.501	0.328	−0.173	Negligible	2.31 × 10^−11^/2.66 × 10^−6^	Yes	Independent correlation
***PROM1***	0.324	0.142	−0.181	Negligible	3.09 × 10^−6^/9.95 × 10^−6^	Yes	Independent correlation
***KRAS***	0.331	0.117	−0.214	Negligible	3.63 × 10^−7^/4.60 × 10^−5^	Yes	Independent correlation
***IL6***	0.295	0.065	−0.230	Negligible	4.67 × 10^−5^/8.38 × 10^−5^	Yes	Independent correlation
***CDK1***	−0.112	−0.367	−0.255	Negligible	1.40 × 10^−5^/3.32 × 10^−7^	Yes	Independent correlation
***VIM***	0.410	0.121	−0.289	Negligible	3.67 × 10^−9^/5.07 × 10^−4^	Yes	Independent correlation
***MYC***	0.294	−0.073	−0.367	Decreased	7.28 × 10^−5^/7.04 × 10^−5^	Yes	Independent correlation
***MKI67***	0.068	−0.312	−0.379	Decreased	1.04 × 10^−3^/1.40 × 10^−5^	Yes	Independent correlation
***CD44***	0.304	−0.118	−0.422	Decreased	1.45 × 10^−6^/1.08 × 10^−4^	Yes	Independent correlation
***CDK2***	0.219	−0.303	−0.522	Decreased	5.16 × 10^−5^/3.93 × 10^−5^	Yes	Independent correlation
***VEGFA***	0.245	−0.282	−0.527	Decreased	1.96 × 10^−4^/2.55 × 10^−4^	Yes	Independent correlation
***SRC***	0.254	−0.296	−0.550	Decreased	6.76 × 10^−3^/6.73 × 10^−5^	Yes	Independent correlation
***AXL***	0.282	−0.270	−0.552	Decreased	6.58 × 10^−6^/2.61 × 10^−5^	Yes	Independent correlation
***SLC2A1***	0.312	−0.300	−0.612	Decreased	2.18 × 10^−6^/1.31 × 10^−4^	Yes	Lower magnitude Δρ
***NOTCH1***	0.342	−0.277	−0.619	Decreased	1.70 × 10^−6^/8.22 × 10^−5^	Yes	Lower magnitude Δρ
***HK2***	0.272	−0.363	−0.636	Decreased	1.08 × 10^−4^/1.62 × 10^−6^	Yes	Lower magnitude Δρ
***TWIST1***	0.276	−0.407	−0.683	Decreased	1.99 × 10^−5^/3.27 × 10^−7^	Yes	Lower magnitude Δρ
***CDH2***	0.282	−0.420	−0.702	Decreased	3.91 × 10^−5^/3.76 × 10^−7^	Yes	Intermediate magnitude Δρ
***TNF***	0.265	−0.440	−0.705	Decreased	6.29 × 10^−5^/2.26 × 10^−9^	Yes	Intermediate magnitude Δρ
***TGFB1***	0.384	−0.337	−0.721	Decreased	4.33 × 10^−8^/2.05 × 10^−6^	Yes	Intermediate magnitude Δρ
***CA9***	0.357	−0.366	−0.723	Decreased	6.57 × 10^−7^/5.67 × 10^−6^	Yes	Intermediate magnitude Δρ
***MMP9***	0.300	−0.432	−0.733	Decreased	6.43 × 10^−5^/7.56 × 10^−9^	Yes	Intermediate magnitude Δρ
***MMP7***	0.419	−0.372	−0.791	Decreased	1.42 × 10^−8^/4.88 × 10^−7^	Yes	Intermediate magnitude Δρ
***MMP2***	0.468	−0.382	−0.849	Decreased	2.05 × 10^−10^/4.82 × 10^−7^	Yes	High magnitude Δρ
***SNAI2***	0.425	−0.431	−0.856	Decreased	1.73 × 10^−9^/1.11 × 10^−8^	Yes	High magnitude Δρ

**Table 3:** Interpretation of the correlation coefficient in biology. Shown is the correlation coefficient range, the medical interpretation, a brief explanation, and an appropriate example.

Correlation Coefficient	Medical Interpretation	Explanation
0–0.2	Poor	No relationship
0.3–0.5	Fair	Real relationship between variables, but it explains only a modest portion of the variation
0.6–0.7	Moderate	Consistent relationship
0.8–0.9	Very strong	Nearly perfect
0.9–1	Perfect	Perfect

**Table 4:** Classification of direction of change and conservative biological associations.

Association	Criteria	Biological Interpretation
Independent correlation	$\left|∆\rho \right|<0.29$	*PROS1*-independent correlation
Strengthened	$\left|∆\rho \right|\geq 0.60$	Enhanced expression in high *PROS1*-expressing tumors
Lower magnitude Δρ	$0.60<\left|∆\rho \right|<0.70$	High *PROS1* tumors exhibit moderately reduced expression
Intermediate magnitude Δρ	$0.70\leq \left|∆\rho \right|<0.80$	High *PROS1* tumors exhibit strongly reduced expression
High magnitude Δρ	$0.80\leq \left|∆\rho \right|<1.00$	High *PROS1* tumors exhibit severely reduced expression

## Data Availability

All data supporting reported results can be found on cBioPortal https://www.cbioportal.org (accessed 1 December 2025).

## Supplementary Materials

The following supporting information can be downloaded at: https://www.mdpi.com/article/10.3390/ijms27072964/s1.
