# Supplementary material for: High Intratumoral PROS1 Expression Correlates with Improved Survival and Is Associated with Suppressed Oncogenic Signaling in Pancreatic Ductal Adenocarcinoma
Source: Int J Mol Sci. 2026 Mar 25;27(7):2964. doi: 10.3390/ijms27072964 (PMC13073249; doi:10.3390/ijms27072964)
Supplement: Supplementary file 1 [file ijms-27-02964-s001.zip › ijms-4165053-supplementary.pdf]

Supplementary Table 1. Results of Cox regression analysis modeling *PROS1* expression as a dose-response relationship.

Cohort	β_1	95% CI	Hazard ratio or Exp(β_1)	95% CI
**TCGA GDC**	-0.0002843	-0.0007051 to 0.0001187	0.9997	0.9993 to 1.000
**TCGA PanCancer**	-0.0004344	-0.001010 to 0.0001047	0.9996	0.9990 to 1.000
**CPTAC Cell 2021**	-0.1411	-0.8568 to 0.5439	0.8684	0.4245 to 1.723

Supplement Table 2. Heterogeneity table of pooled Spearman ρ-values.

	**LOW *PROS1***			**HIGH *PROS1***		
**Gene Name**	**Cochran's Q**	**Cochran's Q p-value**	**I^2 (%)**	**Cochran's Q**	**Cochran's Q p-value**	**I^2 (%)**
**AXL**	0.5799902	0.9009975	0	0.0126621	0.9996225	0
**MERTK**	0.2259198	0.9733003	0	0.0709145	0.995083	0
**TYRO3**	0.743866	0.8628409	0	0.1631218	0.9833109	0
**CA9**	0.6157581	0.8928156	0	0.147074	0.9856437	0
**MKI67**	4.1347485	0.2472765	27.444197	0.1399608	0.9866443	0
**PECAM1**	0.6539092	0.8839876	0	0.6754788	0.8789559	0
**CASP9**	2.5323369	0.4694754	0	0.28164	0.9634437	0
**CD4**	0.9304937	0.8180634	0	0.5804982	0.900882	0
**CD8A**	0.0139564	0.9995633	0	2.4483266	0.484702	0
**STAT3**	0.7730881	0.8558926	0	0.010919	0.9996975	0
**IL1B**	1.1673397	0.7608478	0	0.1028724	0.9914905	0
**CDH1**	1.3177628	0.7249195	0	0.9474427	0.8139663	0
**CDH2**	0.0948923	0.9924432	0	0.2410852	0.9706991	0
**TNF**	0.3634677	0.9476814	0	1.6630544	0.6451786	0
**TGFB1**	0.4028998	0.9396428	0	0.1534265	0.9847325	0
**IL6**	1.4881105	0.6850171	0	5.8530825	0.118981	48.744956
**CASP3**	0.5485613	0.9081003	0	0.0172722	0.9993994	0

**NOTCH1**	0.4956258	0.9198514	0	0.0555256	0.9965776	0
**HIF1A**	0.5863566	0.8995485	0	0.0494001	0.9971227	0
**EPAS1**	0.1907307	0.9790716	0	0.3285448	0.9545735	0
**HIF3A**	0.8354	0.8409824	0	0.2846982	0.9628802	0
**VEGFA**	0.0350648	0.9982719	0	0.1421232	0.9863424	0
**SLC2A1**	1.9256585	0.587979	0	0.4138029	0.9373766	0
**PFKFB3**	0.2233084	0.9737416	0	0.0536143	0.9967509	0
**PFKFB4**	0.2290861	0.9727626	0	0.0966955	0.992231	0
**HK2**	0.8405216	0.8397522	0	0.4204949	0.9359769	0
**PDK1**	1.1982788	0.7534172	0	0.0033551	0.9999484	0
**SNAI1**	0.0970551	0.9921885	0	0.0008365	0.9999936	0
**SNAI2**	1.5710292	0.6659764	0	0.0332857	0.9984009	0
**TWIST1**	0.0673849	0.9954407	0	0.2581456	0.9676978	0
**CD44**	0.6213187	0.8915351	0	3.9400686	0.2680088	23.859193
**PROM1**	0.0098237	0.9997418	0	5.719036	0.1261092	47.543607
**CDK1**	2.6580882	0.4473968	0	0.1888417	0.9793702	0
**CDK2**	0.1558492	0.9843807	0	0.1283476	0.988231	0
**MYC**	0.0264814	0.9988629	0	6.388162	0.094179	53.038135
**E2F1**	1.446273	0.6947248	0	2.2389197	0.5243231	0
**VIM**	0.8577345	0.8356136	0	2.8663076	0.4127005	0
**MMP2**	0.7636053	0.8581509	0	0.1310869	0.9878621	0
**MMP7**	0.0950445	0.9924254	0	1.5909624	0.6614408	0
**MMP9**	0.2183241	0.9745783	0	1.0306154	0.7938445	0
**PIK3CA**	0.9382911	0.8161788	0	1.3317568	0.7216063	0
**AKT1**	0.0252617	0.9989402	0	0.4294171	0.9341008	0
**MAPK1**	1.5366136	0.6738461	0	0.9801081	0.8060651	0
**PTK2**	0.1913297	0.9789767	0	0.1290506	0.9881367	0
**KRAS**	1.8195787	0.6106837	0	4.3309637	0.2278727	30.731353
**NRAS**	0.0570311	0.996439	0	0.0472679	0.9973053	0
**HRAS**	0.2554662	0.9681743	0	0.1923951	0.9788076	0
**SRC**	0.1270204	0.9884085	0	0.2309908	0.9724377	0

Supplement Table 3. Number of patients in each cohort, the age range of patients in the cohort, and the number of female and male patients in each cohort.

**Cohort**	**Number**	**Age Range**	**Sex**	
			**Female**	**Male**

**TCGA GDC**	143	41-84	63	80
**TCGA PanCancer**	142	48-81	64	78
**CPTAC Cell 2021**	112	43-80	54	58
